# Supplementary material for: Illusory object recognition is either perceptual or cognitive in origin depending on decision confidence
Source: PLoS Biol. 2023 Mar 2;21(3):e3002009. doi: 10.1371/journal.pbio.3002009 (PMC10013920; doi:10.1371/journal.pbio.3002009)
Supplement: S1 Text — BF, Bayes factor; HC, high confidence; LC, low confidence. (DOCX) [file pbio.3002009.s001.docx]

**S1 Text: Illusory object recognition is either perceptual or cognitive in origin depending on decision confidence**

Josipa Alilović^1,2^, Eline Lampers^1^, Heleen A. Slagter,^3,4^ & Simon van Gaal^1,2^

*^1^ Department of Psychology, University of Amsterdam, Amsterdam, The Netherlands
^2^ Amsterdam Brain and Cognition, University of Amsterdam, Amsterdam, The Netherlands*

*^3^ Department of Applied and Experimental Psychology, Vrije Universiteit Amsterdam, The Netherlands*

*^4^ Institute for Brain and Behavior, Vrije Universiteit Amsterdam, The Netherlands*

**Supplementary results**

**The time-course of category representations: Sensory localizer - full results**

The 3-way repeated measures ANOVA (factors Latency, Correctness, and Confidence) on decoding scores using the sensory classifier and the occipital-parietal set of electrodes indicated that decoding was overall better for correct than for incorrect decisions (F_1,39_=61.01, p<.001, η_p_^2^=0.61) and for HC versus LC trials (F_1,39_=9.93, p=.003, η_p_^2^=0.203). As in the previous set of analyses using the decision localizer, we found differences in decoding scores across three diagonal time windows (main effect of Latency: F_2,78_=25.56, p<.001, η_p_^2^=0.4), which were modulated by response correctness (Correctness x Latency: F_2,78_=33.93, p<.001, η_p_^2^=0.47) and confidence therein (Latency x Confidence: F_2,78_=5.27, p=.007, η_p_^2^=0.12). We did not find evidence that correctness and confidence in interaction (F_1,39_=0.288, p=.59, η_p_^2^=0.007, BF_excl_=6.67) and a three-way interaction between correctness, confidence, and latency (F_2,78_=1.03, p=.36, η_p_^2^=0.03, BF_excl_=9.502) affected decoding significantly. We continue to unpack these results by performing a repeated measures ANOVA separately for correct and incorrect trials.

For incorrect trials, decoding differed among three diagonal time-windows (F_2,78_=5.91, p=.004, η_p_^2^=0.13), but the evidence for a modulation by confidence was weak (Latency x Confidence: F_2,78_=2.25, p=.11, η_p_^2^=0.06, BF_excl_=2.49). The main effect of confidence was not robust overall (F_1,39_=3.23, p=.08, η_p_^2^=0.08, BF_01_=1.86, **Fig 4**). Next, we performed a set of planned comparisons examining decoding in each window of interest as a function of confidence. In the earliest time-window (80-130 ms) decoding was significantly below chance for both confidence levels (LC: t_39_=-3.4, p=.002, d=-0.54; HC: t_39_=-3.91, p<.001, d=-0.62). We found that decoding was below chance in the following 150-200 ms time-window, in particular for LC trials (LC: t_39_=-3.04, p=.004, d=-0.48; HC: t_39_=-0.11, p=.91, d=-0.02, BF_01_=5.83). We did not find evidence for late decoding stage (350-500 ms) for either confidence levels (LC: t_39_=0.16, p=.88, d=-0.03, BF_01_=5.8; HC: t_39_=1.27, p=.21, d=0.2, BF_01_=2.8). Thus, early sensory stages represented the veridical stimulus category, while category representations of misreported stimuli could not be decoded at any stage along the diagonal.

For correct trials, decoding scores depended on the decoding latency (F_2,78_=48.17, p<.001, η_p_^2^=0.55), confidence (F_1,39_=11.96, p<.001, η_p_^2^=0.24) and their interaction (Latency x Confidence: F_2,78_=7.77, p<.001, η_p_^2^=0.17). Decoding was above chance for both confidence levels in the early and mid-stage (all p’s <.001, see **Fig 4C**), but there was no evidence for decoding in the late diagonal stage (LC: t_39_=1.104, p=.28, d=0.18, BF_01_=3.33; HC: t_39_=1.34, p=.19, d=0.21, BF_01_=2.55). Again, this was unsurprising, given that a decision was not required in the sensory localizer task. In line with the previous analyses, we only found evidence that the mid-decoding window (150-200 ms) was modulated by confidence (LC vs. HC: t_39_=-5.2, p<.001, d=-0.82, all other p’s>.23, BF_01_>2.97). Thus, as expected, these results suggest that early sensory stages of processing represented the presented stimulus category both on trials in which the stimulus category was later correctly and incorrectly reported, while the 350-500 ms late-stages decoding did not contain any categorical information specific for either the presented or reported stimulus.

Confirming our previous results, during the perceptual maintenance stage, we decoded the incorrectly reported stimulus category when participants reported high confidence in their decision, but we did not find evidence for this in LC trials (LC: t_39_=1.78, p=.08, d=0.28; BF_01_=1.4; HC: t_39_=3.03, p=.004, d=0.48, BF_01_=0.12, decoding in LC vs. HC: t_39_=-1.94, p=.06, d=-0.31, BF_01_=1.07, **Fig 4E**). Sensory classifiers were not sensitive to decision processes (notice the absence of the late decoding stage in **Fig 4C**), which thus eliminates the possibility that this effect was decision-related. On correct trials, during the perceptual maintenance stage, the activity patterns reflected the veridical stimulus category, irrespective of reported confidence (decoding in low vs. high-confidence trials: t_39_=-0.98, p=.34, d=-0.15, BF_01_=3.76, **Fig 4D-E**).

Note that cluster-based permutation test on each sample of off-diagonal decoding scores for correct and incorrect report trials (**Fig 4C-D**) suggested that early classifiers (150-200 ms) generalized significantly to even earlier latencies than 350 ms. For instance, as shown on **Fig 4D**, decoding was significantly above chance for high-confidence trials already from ~210-300 ms post-stimulus, starting again at around 370 ms. Early latencies to which 150-200 ms sensory-tuned classifiers generalized, further corroborate the conclusion that we were indeed observing a perceptual effect.

**Decision localizer: all electrodes**

We also examined cross-task decoding as a function of decoding latency (early, mid, late), decision correctness (correct/incorrect) and confidence (low confidence (LC): level 1 and 2 versus high confidence (HC): level 3-4) using the decision localizer and all electrodes **(S4 Fig)**. Testing labels in the main discrimination task were based on the decision of the subject (being face or house), not the actually presented stimulus.

The omnibus ANOVA with factors Latency, Confidence and Correctness indicated that decoding was overall better for correct versus incorrect trials (F_1,39_=137.89, p<.001, η^2^=0.78) as well as for HC versus LC trials (F_1,39_=18.48, p<.001, η^2^=0.32). Decoding differed across the three decoding stages (F_2,78_=30.18, p<.001, η^2^=0.44), but this depended on the trial correctness (Correctness x Latency: F_2,78_=38.62, p<.001, η^2^=0.5) and decision confidence (Confidence x Latency: F_2,78_=5.37, p=.007, η^2^=0.12). We were unable to find evidence that decoding was affected by an interaction between correctness and confidence (F_1,39_=0.094, p=.76, η_p_^2^=0.002, BF_excl_=6.39) or the three-way interaction between correctness, confidence, and latency (F_2,78_=0.61, p=.55, η_p_^2^=0.02, BF_excl_=10.322).

The follow-up repeated measures ANOVA with factors Latency (early, mid, late) and Confidence (low, high), separately for correct and incorrect trials, yielded largely similar results to those reported in the main text using the occipital-parietal electrode set. For correct trials, decoding was better for HC than for LC trials (F_1,39_=30.97, p<.001, η^2^=0.44). Decoding differed across three diagonal stages (F_2,78_ =51.25, p<.001, η^2^=0.57), also in interaction with confidence (Confidence x Latency: F_2,78_=6.81, p=.002, η^2^=0.15). In each but the earliest processing stage, decoding was modulated by confidence and was better for HC than LC trials (early stage: t_39_=-1.83, p=.075, d=-0.29, BF_01_=1.29; mid-stage t_39_=-5.38, p<.001, d=-0.85; late stage: t_39_=-3.27, p=.002, d=-0.52). We also tested the off-diagonal decoding stage reflective of the perceptual maintenance of category information. There too, decoding was better for HC versus LC trials (t_39_=-3.22, p=.003, d=-0.51). Decoding on HC and LC trials in all three diagonal stages and in the off-diagonal perceptual maintenance stage was significantly different than chance (all p’s > .001).

On incorrect trials, decoding was modulated by confidence (F_1,39_=5.79, p=.021, η^2^=0.13), latency of the diagonal decoding stage (F_2,78_=10.81, p<.001, η^2^=0.22), but we did not find evidence for their interaction (Latency x Confidence: F_2,78_=2.27, p=.11, η^2^=0.06, BF_excl_=2.21). Decoding on HC and LC trials in the earliest 80-130 ms window did not differ (t_39_=-0.19, p=0.854, d=-0.03, BF_01_=5.77), but it was in both cases significantly below chance (LC: t_39_=-5.34, p<.001, d=-0.85, HC: t_39_=-2.28, p=.028, d=-0.36). This suggested that the earliest stage of visual information processing represented the veridical stimulus category, irrespective of confidence. Decoding was also below chance for incorrect trials in 150-200 ms time-window when participants expressed low confidence in their decision (t_39_=-6.39, p<.001, d=-1.01), but we did not find evidence that this was the case when they were highly confident (t_39_=-0.604, p=.55, d=-0.1, BF_01_=4.49; HC vs. LC: t_39_=-2.77, p=.009, d=-0.44). In the later 350-500 ms window, we found the opposite decoding pattern. Above chance decoding was observed in particular for HC trials (LC: t_39_=-1.44, p=.16, d=-0.23, BF_01_=2.27; HC: t_39_=2.07, p=.045, d=0.33), which also significantly differed from LC trials (t_39_=-2.72, p=.01, d=-0.43).

Decoding was significantly above chance in the late off-diagonal perceptual maintenance stage, however, again in particular for high-confidence trials (LC: t_39_=1.06, p=.296, d=0.17, BF_01_=3.48; HC: t_39_=2.36, p=.024, d=0.37), but evidence for the difference between confidence levels was marginal (t_39_=-1.87, p=.07, d=-0.3, BF_01_=1.2). These findings suggest that when participants misreported a stimulus category, neural activity patterns reflected the veridical stimulus category during early and mid-latencies, however, specifically on LC trials in the mid-stage (150-200 ms), thus when participants were uncertain in their decisions. Later in time (350-500 ms) and specifically when participants were confident in their decision, mental representations reflect the misreported stimulus category. These results are thus consistent with the results presented in the main text.

**Sensory localizer: all electrodes**

Using all electrodes, decoding was also examined as a function of decoding latency (early, mid, late), decision correctness (correct/incorrect) and confidence (low confidence (LC): level 1 and 2 versus high confidence (HC): level 3-4) based on the sensory localizer task (**S5 Fig**). Testing labels in the main discrimination task were again based on the decision subjects made (being face or house), not the actually presented stimulus. Overall, the results are consistent with the results presented in the main text, obtained on the occipital-parietal electrode set.

The omnibus ANOVA indicated that decoding was overall better for correct versus incorrect trials (F_1,39_=53.48, p<.001, η^2^=0.58). Decoding was also overall better for HC versus LC trials (F_1,39_=8.75, p=.005, η^2^=0.183). Decoding also differed depending on the decoding latency (F_2,78_=21.51, p<.001, η^2^=0.36). Consistent with previous analyses, correctness and confidence interacted with decoding latencies (Correctness x Latency: F_2,78_=30.49, p<.001, η^2^=0.44; Confidence x Latency: F_2,78_=5.07, p=.009, η^2^=0.12). We did not find evidence for an interaction between correctness and confidence (F_1,39_=0.13, p=.73, η_p_^2^=0.003, BF_excl_=6.14) nor a three-way interaction between correctness, confidence and latency (F_2,78_=0.17, p=.85, η_p_^2^=0.004, BF_excl_=12.89).

On correct trials, decoding was higher for HC versus LC trials (F_1,39_=14.02, p<.001, η^2^=0.26), and confidence further modulated differences in decoding across diagonal decoding stages (main effect of Latency: F_2,78_ =48.65, p<.001, η^2^=0.55; Confidence x Latency: F_2,78_=8.65, p<.001, η^2^=0.18). In the early and mid-stage, decoding was above chance for both confidence levels (all p’s <.001, see **S5A Fig**). The late-late stage decoding for both confidence levels was also different than chance, but it was not as robust (LC: t_39_=2.3, p=.03, d=0.36; HC: t_39_=2.28, p=.03, d=0.36). During the perceptual maintenance stage, the activity patterns as well reflected the veridical/reported stimulus category (both p’s <.001), and we found no evidence that this depended on the level of confidence (LC vs. HC: t_39_=-0.98, p=.34, d=-0.15, BF_01_=3.76).

On incorrect trials, decoding did not differ substantially between HC and LC trials (F_1,39_=2.01, p=.165, η^2^=0.05, BF_01_=2.07) and also not across diagonal decoding stages (F_2,78_ =0.96, p=.39, η^2^=0.024, BF_01_=7.94). We also found no evidence that decoding performance was modulated by confidence in interaction with decoding latency (Confidence x Latency: F_2,78_=1.28, p=.28, η^2^=0.03, BF_excl_=5.88). The planned comparisons per confidence level across diagonal decoding stages revealed that the veridical stimulus category on incorrect trials could be decoded in particular on low confidence trials in the early (LC: t_39_=-4.46, p<.001, d=-0.71; HC: t_39_=-1.5, p=.14, d=-0.24; LC vs. HC: t_39_=-0.56, p=.58, d=-0.09) and mid-stage (LC: t_39_=-3.94, p<.001, d=-0.62; HC: t_39_=-0.32, p=.75, d=-0.05; LC vs. HC: t_39_=-2.18, p=.04, d=-0.35, thus reflected in negative decoding scores). Late-stage decoding was not different than chance on LC (t_39_=-1.23, p=.23, d=-0.19) and HC (t_39_=-0.24, p=.81, d=-0.04; LC vs. HC: t_39_=-0.28, p=.78, d=-0.045) trials. However, importantly, during the perceptual maintenance stage we could decode the incorrectly reported stimulus category when participants reported high confidence in their decision, similarly to the results reported in the main text (LC: t_39_=0.26, p=.79, d=0.04; BF_01_=5.67; HC: t_39_=2.64, p=.01, d=0.42; LC vs. HC: t_39_=-2.35, p=.02, d=-0.37, **S5C Fig**).
