# Supplementary figures and images for: Illusory object recognition is either perceptual or cognitive in origin depending on decision confidence
Source: PLoS Biol. 2023 Mar 2;21(3):e3002009. doi: 10.1371/journal.pbio.3002009 (PMC10013920; doi:10.1371/journal.pbio.3002009)

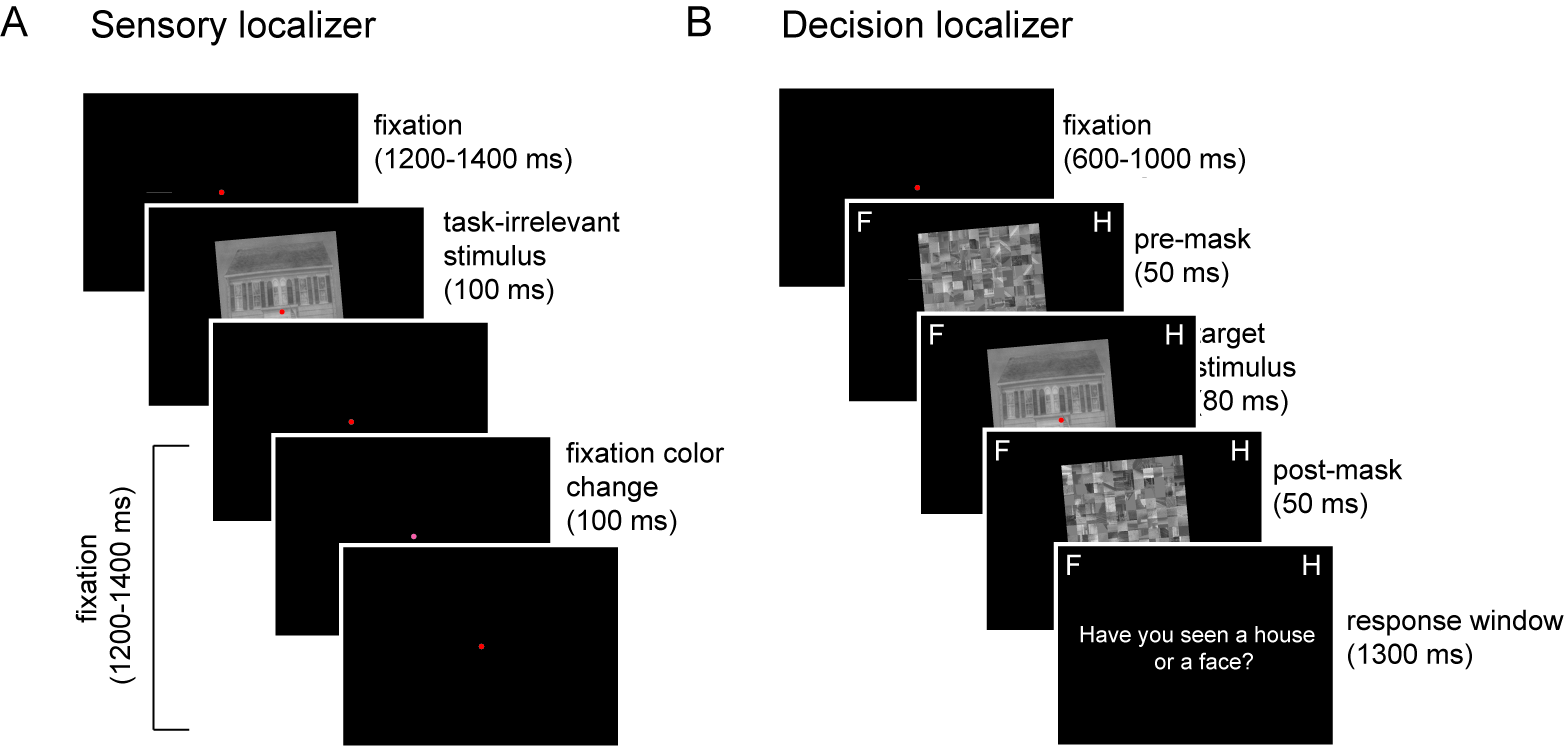

Supplement: S1 Fig — (A) Each trial of the sensory localizer task started with a central red fixation dot for 1,200–1,400 ms, during which an infrequent contrast change of the dot could happen (20% of trials). Participants needed to press the spacebar as soon as they noticed the contrast change. In the same time interval, a house or a face image was briefly shown on the screen, which needed to be ignored. Images were either tilted to the right or to the left (task-irrelevant feature) at a 5° or 355° angle. Note that in the example trial, only a left-tilted house image is shown. (B) Each trial of the decision localizer task started with a central red fixation dot after which a forward mask was shown, followed by an image of a face or a house and a backward mask. Images were either tilted to the right or to the left (task-irrelevant feature) at a 5° or 355° angle. On every trial, participants reported whether they perceived a house or a face and indicated their confidence in this decision. (TIF) [file pbio.3002009.s002.tif]

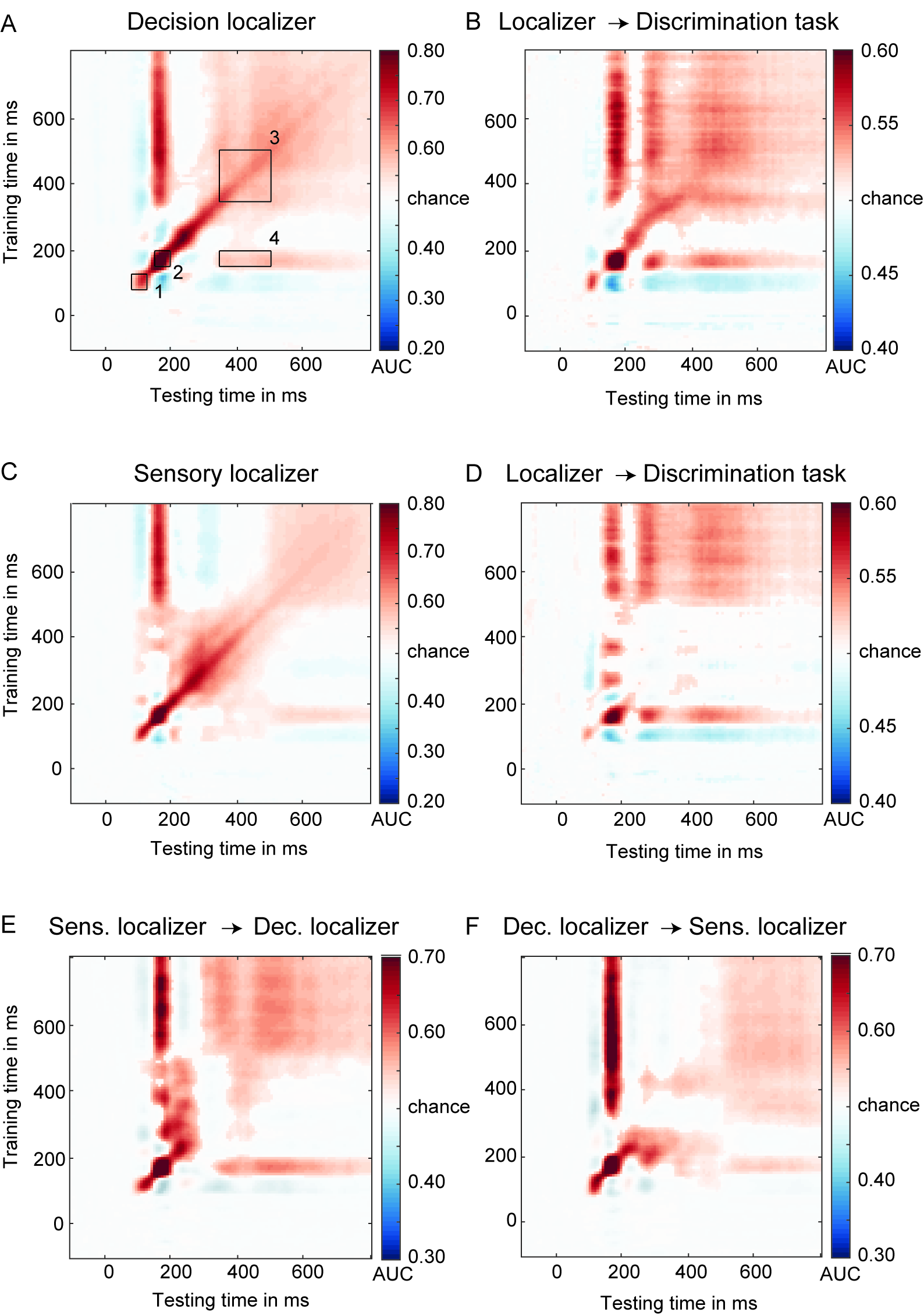

Supplement: S2 Fig — (A) GAT matrix for the decision localizer and regions of interest marked by inset black boxes (numbers 1–4). (B) Classifiers trained on the decision localizer applied to the main discrimination task (cross-task validation procedure). (C) GAT matrix for the sensory localizer. (D) Classifiers trained on the sensory localizer applied to the main discrimination task (cross-task validation procedure. (E) Classifiers trained on the sensory localizer applied to the decision localizer (cross-task validation procedure). (F) Classifiers trained on the decision localizer applied to the sensory localizer (cross-task validation procedure). All GAT matrices are based on occipital–parietal electrodes. The underlying data and scripts supporting this figure can be found on FigShare (https://doi.org/10.21942/uva.c.6265233.v1). (TIF) [file pbio.3002009.s003.tif]

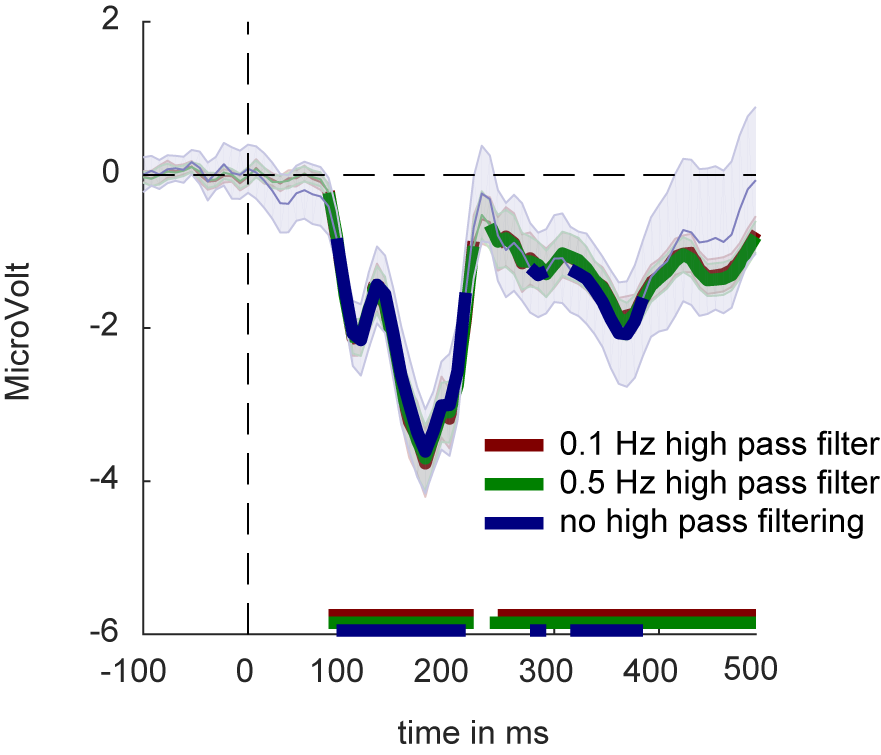

Supplement: S3 Fig — ERPs are time-locked to stimulus onset in the passive localizer with 3 different high-pass filtering settings. The region of interest plotted consists of the following electrodes: O1, O2, PO3, PO4, PO7, PO8. The underlying data and scripts supporting this figure can be found on FigShare (https://doi.org/10.21942/uva.c.6265233.v1). (TIF) [file pbio.3002009.s004.tif]

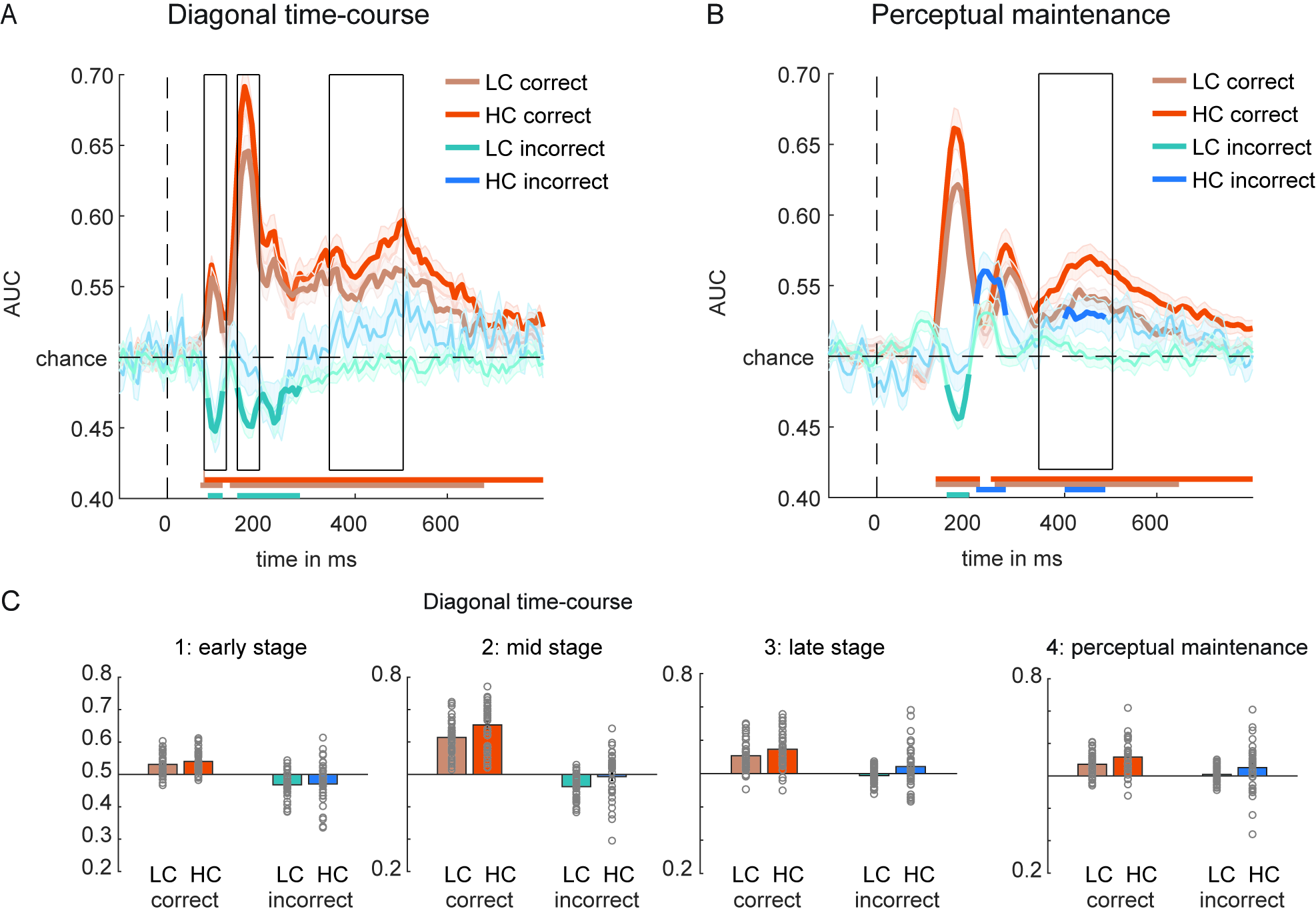

Supplement: S4 Fig — (A) On-diagonal time courses of correct and incorrect decisions, separated by confidence (LC, low confidence; HC, high confidence). Black boxes indicate the time-windows (early, mid, late) used for averaging the AUC scores. (B) Perceptual maintenance is derived by training a classifier on the time-window 150–200 ms and testing it across the entire time-window. In (A) and (B), colored horizontal lines indicate periods of significant decoding with respect to chance. Classification performance was evaluated at each time point using cluster-based permutation testing (two-tailed cluster-permutation, alpha p < 0.05, cluster alpha p < 0.05, N permutations = 1,000). (C) Bar plots showing average AUC values for the time-windows of interest highlighted in panels (A) and (B). The underlying data and scripts supporting this figure can be found on FigShare (https://doi.org/10.21942/uva.c.6265233.v1). (TIF) [file pbio.3002009.s005.tif]

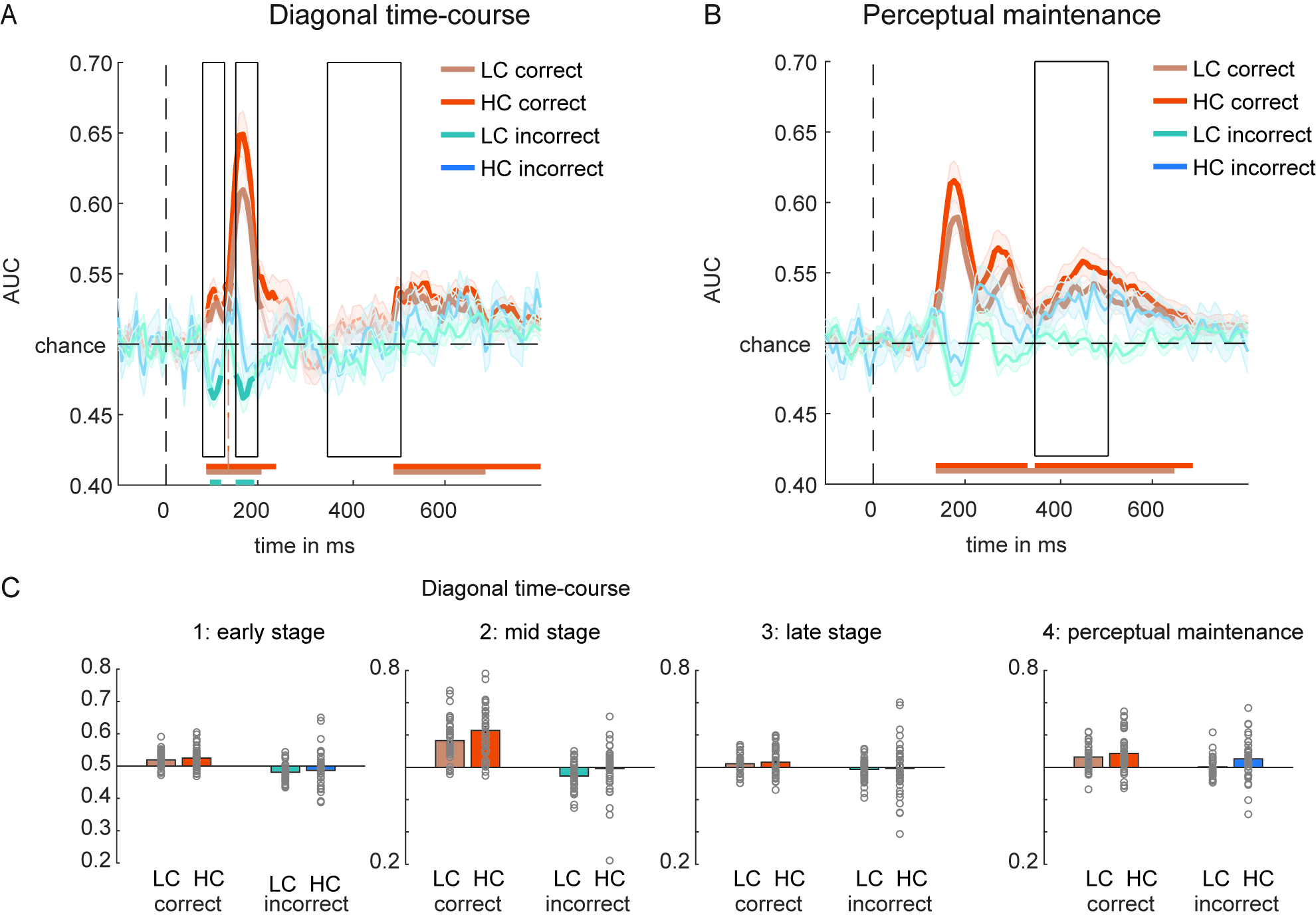

Supplement: S5 Fig — (A) On-diagonal time courses of correct and incorrect decisions, separated by confidence (LC, low confidence; HC, high confidence). Black boxes indicate the time-windows (early, mid, late) used for averaging the AUC scores. (B) Perceptual maintenance is derived by training a classifier on the time-window 150–200 ms and testing it across the entire time-window. In (A) and (B), colored horizontal lines indicate periods of significant decoding with respect to chance. Classification performance was evaluated at each time point using cluster-based permutation testing (two-tailed cluster-permutation, alpha p < 0.05, cluster alpha p < 0.05, N permutations = 1,000). (C) Bar plots showing average AUC values for the time-windows of interest highlighted in panels (A) and (B). The underlying data and scripts supporting this figure can be found on Fig Share (https://doi.org/10.21942/uva.c.6265233.v1). (TIF) [file pbio.3002009.s006.tif]
