# Supplementary material for: Illusory object recognition is either perceptual or cognitive in origin depending on decision confidence
Source: PLoS Biol. 2023 Mar 2;21(3):e3002009. doi: 10.1371/journal.pbio.3002009 (PMC10013920; doi:10.1371/journal.pbio.3002009)
Supplement: Attachment — Submitted filename: Rebuttal_Reviews PLOS Bio.docx [file pbio.3002009.s007.docx]

Amsterdam, October 28th, 2022

Dear Editor,

We would like to express our gratitude towards the reviewers and editors of PLOS Biology for the swift revision process and clear commentaries on our manuscript. We have addressed the comments raised by the reviewers and revised our manuscript extensively. Specifically, we have created new figures for all results and provided additional interpretation and discussion of our results. We have also provided more details about methods and analyses protocols. With these revisions we believe the quality of the manuscript has significantly improved.

Please see below our detailed responses (in blue) to the reviewer comments (in black). Substantial revisions have been tracked in the renewed manuscript and new sections or major changes have been added to this letter as well (inset text).

We hope that we have addressed the reviewers’ remarks to satisfaction and hope you will now find the manuscript suitable for publication in PLOS Biology.

Yours sincerely, and on behalf of all co-authors,

Simon van Gaal

Reviewers' comments

**Reviewer. 1: Guillaume Rousselet - note that this reviewer has signed his review**

This is a very good article, presenting an ambitious and clever experiment, with thoughtful analyses. I have a lot of relatively minor comments and suggestions to try to improve the presentation, including illustrations and reporting.
We thank the reviewer for these kind words and the valuable comments and suggestions.

\## Paradigm
To check that the localisers did differ as intended, it would be useful to illustrate, maybe in Figure 1, cross-classification results: classification of localiser 1 data using the classifier trained with localiser 2 data, and vice versa.
Thanks for this suggestion. We have run the suggested analysis and report the results in supplementary figure S2. We would like to note that the decision localizer was identical to the main task and therefore the results look very similar to figure 4B of the paper. Therefore, we felt these additional analyses/result would better be suited for the supplementary information. Based on this, one can conclude that the localisers indeed differed as intended.

\## Illustrations
Figures 1-4: please replace the bar graphs with scatterplots and remove the little star system. P values are not measures of effect sizes or the importance of the results. If the goal is to emphasise effect sizes, it would be more informative to plot distributions of pairwise differences. This is essential to let readers assess how many participants show the effects, and the range of effect sizes. See suggestions here: <https://onlinelibrary.wiley.com/doi/full/10.1111/ejn.13400>
Thanks for pointing this out. We have followed your suggestion and included scatterplots in all our figures together with the bar graphs for each condition. All figures have been updated accordingly. We have also removed the little star system throughout the paper.

Figures 2-4: please increase font size. Would be worth splitting the content of figures 2 and 3 across 2 figures to increase the size of all panels. Figure 4 could do with having 2 rows.
Thanks for the suggestions. We have increased the font size of all the figures and we have also adapted figure 4 in line with the reviewer’s suggestion. Because of other comments made by the reviewer, the sizes of the different panels of figures 2 and 3 have changed and increased and we have splitted figure 2 across two separate figures (now Figure 2 and 3). Especially the panels showing the line plots from the decoding analysis have increased considerably in size. We believe the new lay-out and size of these figures has improved the readability of the figures.

"As shown in Fig. 2E and 2G, diagonal decoding profiles were very different for correct and incorrect decisions" - this would be better reported by adding a new plot with correct and incorrect time-courses superimposed, and plotted separately for low and high confidence. Interactions could also be illustrated by plotting time-courses of differences.

Thanks for the suggestions. As suggested, we have superimposed the line plots of the correct and incorrect decisions (also based on comments by the other reviewers). After a few iterations of plot design and panel ordering, we have decided to plot the four conditions (correctness (2) x confidence (2)) in one panel, because that clearly shows the comparisons of interest at the same scale (see for a related comment about scales, minor comments of R3). We did not want to increase the size of the figures even more, so we have omitted time course differences, for two reasons. First, the figures became too cluttered and therefore the individual lines were difficult to isolate. Second, because all the relevant comparisons (differences) can be observed in the bar graphs (single subject data points included), which have now been re-ordered based on the reviewer’s comments. We have now plotted the correct and errors trials in one panel, separately for the different stages for better comparison. Finally, because the most interesting data pattern relates to the interaction of confidence and correctness, difference plots (e.g., subtracting errors from corrects) made the relevant/interesting data pattern more difficult to interpret.

I find this bit difficult to understand: "Most prominently, it can be observed that for correct trials, all processing stages show positive decoding, whereas in sharp contrast the two early stages for the incorrect trials showed negative decoding. This negative decoding pattern on incorrect decisions illustrates that early EEG data patterns look more similar to the presented stimulus category than to the reported stimulus category (note again that the decisions were used as the classifier labels)." By negative decoding, you mean below chance, not negative values? More importantly, could you provide a toy example, with simulated data from one participant, that would clearly demonstrate this dissociation and support your interpretation? Or is there a reference about this type of classifier's behaviour?

Thanks for pointing this out. Indeed, negative decoding reflects “below chance decoding”. Below chance decoding in this study means that the classifier finds evidence in the data for the opposite category than the label used for that class in a two-class decoding scheme (e.g. a face is reported, but the classifier finds evidence for house decision). In our case, for example, before approximately 200 ms, the classifier scores below chance on high confidence trials. Because we used the decision of the participant as the classifier label (e.g., face) and because on incorrect decisions the presented stimulus is always from the other category (e.g., house), this leads to below chance decoding in trials with incorrect decisions. In other words, the classifier finds most evidence for house, the stimulus, and less so for face, the decision, before 200 ms after stimulus presentation. Therefore, we believe that negative decoding better captures the phenomenon that the classifier finds negative evidence for the decision category. We have clarified this better in the revised manuscript and we now explicitly introduce the terms *positive* and negative decoding, and clarify that this is meant to reflect decoding scores relative to chance level.

Modified/added on page 6:

Note that the “testing labels” of the classifier were based on the decision of the subject (being face or house), not the presented stimulus. Obviously, for correct trials the presented stimulus and the decision are by definition the same. For incorrect trials however, the stimulus and the decision are of different categories. This means that if decoding scores are observed that are higher than chance level, the underlying data pattern correlates with the category of the reported stimulus, the decision. We will refer to this situation as positive decoding (with respect to chance level). In contrast, when below chance level decoding is observed, this would reveal that the underlying data pattern correlates with the presented stimulus, not with the reported stimulus (they are different on incorrect trials, see Methods for details). The classifier thus finds negative evidence for the decision, and therefore we will refer to this situation as negative decoding (with respect to chance level).

\## Stat reporting.
The results section could be improved by going beyond outdated statements about the statistical significance of effects. Here are resources to more carefully report frequentist results, without falling into the trap of artificial dichotomies:

Language for communicating frequentist results about treatment effects
<https://discourse.datamethods.org/t/language-for-communicating-frequentist-results-about-treatment-effects/934>

Scientists rise up against statistical significance
<https://www.nature.com/articles/d41586-019-00857-9>

Inferential Statistics as Descriptive Statistics: There Is No Replication Crisis if We Don't Expect Replication
<https://www.tandfonline.com/doi/full/10.1080/00031305.2018.1543137>

Abandon Statistical Significance
<https://www.tandfonline.com/doi/full/10.1080/00031305.2018.1527253>

Thanks for these suggestions. We have gone over the entire manuscript with the aim to more carefully report frequentist result.

"Sensitivity (d') did not differ significantly from zero" - this suggests an equivalence test was performed. Otherwise, you can only conclude that you reserve judgement or that you did not get enough evidence. A simple statement would just state the results and point to a detailed graphical representation. There is no need to dichotomise all p values, unless you're trying to localise in space or time using brain imaging data - hard to see how to do that without some threshold.

We agree with the reviewer that the phrasing was not careful enough here. We have decided to remove the statistics of the individual confidence levels, because these are in fact not crucial. The relevant analysis regards the increase in sensitivity (d’) as a factor of decision confidence. The results section has been updated accordingly (see page 4). We have also updated the figure and have included individual datapoints of participants.

In this sentence and possibly others, you seem to imply that the lack of evidence for an effect is evidence for its absence: "This was only the case when participants were highly confident in their incorrect decision (HC: t39=3.86, p<.001, d=0.61; LC: t39=1.41, p=.17, d=0.22, BF01=2.35; LC vs HC: t39=-3.06, p=.004, d=-0.48; Fig. 2H)". The statement should be revised by drawing attention to effect sizes and a supporting illustration. The absence of an effect cannot be derived from p values.

Thanks for pointing this out. We have rephrased this specific sentence, as well as other sentences, where this was the case.

Same issue in lines 307-310 as well as other places, including supplementary information:
"Decoding strength was equal for correct and incorrect decisions (F1,39=0.06, p=.82, ηp2=0.001, BF01=5.7) and confidence did not modulate this decoding profile in any way (F1,39=0.54, p=.47, ηp2=0.014, BF01=4.77; interaction correctness x confidence: F1,39=0.18, p=.67, ηp2=0.005, BFexcl=4.22)."

Lines 270-272: can you provide values to qualify "much weaker"? For instance median difference + range?
We have gone over the entire manuscript and have improved the statements about (in)significance, including the ones highlighted above. We have plotted individual data points for all 40 subjects to highlight the range of data in all bar plots.

275: "all other effects were identical" -> "were similar", as they cannot be identical.

Thanks, this was changed.

808: please add a justification for your alpha.
We have added the following justification to the revised MS and referenced the Lakens et al. Nature Human Behavior paper (1) (“justify your alpha”) for context.

*Added on page 24*

We took a frequentist statistical approach and chose an alpha of p<.05 as the threshold for significance as is typical, to guard against false positives. Note, however, that this threshold is relatively arbitrary, and that frequentist statistics cannot provide evidence for the null hypothesis (absence of an effect) (99). Therefore, we also report effect sizes, and in case of non-significant effects as indicated by p>.05, followed up with a Bayesian equivalent of the same test in order to quantify the strength of evidence for the null hypothesis (H0). By convention, Bayes factors from 1 to 3 were considered as anecdotal, 3 to 10 as substantial, and those above 10 as strong evidence in favor of H0.

*Added on page 26*

Besides our hypothesis driven analyses based on our predefined selection of time-windows, we additionally statistically compared each time point to chance (both for on- and off-diagonal decoding) by applying group-level permutation testing with cluster correction for multiple comparisons. We employed two-tailed cluster-permutation testing with an alpha of p<.05 and cluster alpha p<.05 (N permutations=1000, as implemeted in ADAM toolbox). These latter analyses were mainly performed to evaluate whether our hypothesis driven analyses may have missed potentially relevant effects, and if so, we recommend future studies to replicate these effects to evaluate the robustness of them ((1)).

\## Other points
"To determine the nature of misreports, we present a novel way to dissociate sensory from post-sensory or decision-related processes during human perceptual decision-making using electroencephalography (EEG) in combination with multivariate pattern analyses (MVPA)." I'm struggling to see the novelty in the approach, so maybe you need to be more specific. These articles for instance, contain very similar ingredients:
<https://jov.arvojournals.org/article.aspx?articleid=2121325>
<https://elifesciences.org/articles/68491>
<https://academic.oup.com/cercor/article/16/4/509/382221>
<https://www.jneurosci.org/content/26/35/8965>
<https://www.pnas.org/content/106/16/6539.short>
Using EMG could also be a way to assess the motor stage:
<https://hal.archives-ouvertes.fr/hal-01292369/document>
In addition to refs 63-64, this is a good paper about mapping decoding results:
<https://www.sciencedirect.com/science/article/pii/S1053811905003381>
We appreciate the comments made about the novelty of the methods and the references that are suggested by the reviewer. We have added all of the suggested references to the revised manuscript and have better specified, as well as down toned, the novelty of the methodological approach. We believe the paper has really improved because of the added references and context.

"N170 ERP component, specific to face processing" -- that component is also observed for letters, words, and biological motion for instance, so not specific to faces.
Thanks for pointing this out. We have rewritten this sentence and provided appropriate references.

*Added on page 4*

The timing (150–200 ms; the decoding peak was found at ~166 ms) and scalp topography of the early peak is probably related to the N170 ERP component, often related to face processing (38–40), although also observed for letters, words and biological motion (41, 42).

"In general, activity observed in sensory regions can thus be a mixture of both feedforward and feedback processes, especially at longer latencies (>~100 ms) (39)". Not sure if this has been confirmed and if it applies to non-moving stimuli, but feedback seems to affect the earliest V1 responses:
<https://pubmed.ncbi.nlm.nih.gov/11152714/>
See also this in humans:
<https://pubmed.ncbi.nlm.nih.gov/11797091/>
Both reinforcing your point, that indeed activity in sensory regions is a mixture of feedforward and feedback processes.
Thanks for the suggested references, which have been added to the revised manuscript at the suggested location.

"were viewed with a distance of 90 cm from the monitor" - please indicate if a chin rest was used. If not, adjust text to something like "unconstrained viewing distance of approximately 90 cm".
A chin rest was used. We have adjusted the text accordingly.

663-5: "Furthermore, letters F and H, for faces and houses respectively, were shown on every trial in the right and left upper corner, or vice versa, depending on the block, as a as a reminder of the response mapping to the button press." For replicability, information is missing about the size, position and duration of these stimuli.
Thanks for pointing this out to us. We included the missing information in the Methods section on page 22.

711-3: "EEG data was re-referenced to the average of the earlobes, high-pass filtered at 0.5 Hz and low-pass filtered at 40 Hz." Information is missing about the type of filters. Please also provide a figure illustrating filter distortions, ideally using a synthetic signal:
<https://www.sciencedirect.com/science/article/pii/S0165027012002361>
<https://www.sciencedirect.com/science/article/pii/S0165027021000157>
A gentle 0.5 Hz HP filter is unlikely to severely distort your temporal estimates, but it is worth checking and reminding readers about the dangers of HP filtering.
We used the standard filtering protocol implemented in EEGlab (pop_eegfiltnew.m) with default filter settings. In the default filter settings, the filter order is: 2 * cutoff frequency for highpass (for cutoff < 2Hz) and 25% of cutoff frequencies for lowpass. In this function, if high-pass and low-pass cutoff frequencies are both selected, low-pass and high-pass parts will have the same slopes. Frequently, the low-pass slope is therefore steeper than necessary. To avoid this problem, EEGlab recommends first applying the low-pass filter and then, in a second call, the high-pass filter. This is exactly what we did. We have added a figure in the supplements (S3) showing the ERPs for a occipto-temporal cluster of electrodes where decoding weights were strong for three filtering settings: no high-pass filtering, 0.1 Hz high pass filtering and 0.5 Hz high-pass filtering (used in this study). What can be observed is that the signal timing is virtually identical. We have added a cautionary note in the supplements about HP filtering and also cited the suggested papers on page 23.

719: "All analyses were done using custom scripts..." - this would be a good place to mention that all the code and data are available online.
Thanks, we have this information to a new “data availability” paragraph on page 26.

\## Writing
The section starting line 101, "The time-course of category representations: Decision localizer", is difficult to read. It would help to refer to the little squares 1-4 in the figure. The squares are mentioned in a subsequent paragraph, but that description should be integrated with the earlier description. I would make figure 2A much larger and stand alone, to more carefully guide readers through it. Views of the diagonal, like in panels E and G are very useful, and would help support the text description and the understanding of panel A.
Thanks for the suggestions. We have now rewritten several paragraphs on page 4-5 with the goal to make them clearer and to introduce our decoding strategy better. We mention the squares now more explicitly early on and we have also made figure 2A and 2B much larger and stand alone, as suggested. We agree with the reviewer that this improves the readability a lot, thanks.

"this neural representation "flipped" later in time" - avoid the quotation marks of mystery, be specific instead. Same for "global ignition" and other expressions, unless they are quotes.

Thanks, fixed.

This sentence is hard to read:
"Here we investigated this issue and examined at what level of analysis visual stimuli that were misreported (e.g., face presented, house reported) were actually misrepresented, at the neural level, aiming to dissociate sensory from decision-related explanations."
Suggested revision:
"Here we investigated this issue and examined if misreported visual stimuli (e.g., face presented, house reported) were misrepresented at the sensory or the decision stage."
Thanks, adjusted according to the suggestions, see page 2.

"As can be seen in Fig. 1, the orientation of the presented images was either left tilting or right tilting, with 50/50 likelihood." In Figure 1 all the images are tilted to the left.
Thanks for noting this, we fixed it. We have added a statement to the legend of the figure noting that in panel A only left-tilted images are shown.

Lines 108-109: "In contrast, generalization across time analyses can" - delete "analyses"? That whole sentence needs to be rephrased to remove the repetition "generalization across time [...] observed when a classifier trained on certain time points generalizes to other data points earlier or later in time".
Done.

Line 112: "Category-specific neural representations could be robustly decoded" -- robust to what? I would delete "robust".
Done.

Figure 2 caption: "On the right covariance/class separability" - is a comma missing after "right"?
Yes indeed, fixed.

Line 245: "despite never been objectively presented and never been" -> "despite never being presented and never being..." Not sure what an "objective presentation" means. Would be clearer to state "presented on the screen".
Fixed accordingly.

803: "covariance/class separability map maps were" - extra "map"?
Fixed.

Signed review: Guillaume Rousselet

**Reviewer 2:**
The manuscript is a report of an EEG experiment where participants were asked to discriminate images of faces and houses near threshold. Multivariate analyses are performed and reveal that early sensory processing is consistent with the physical stimulus whereas later processing is consistent with the percept. The experiment is cleanly designed and analyses, and the manuscript is well-written. But I am not convinced that the results are sufficiently ground-breaking to appear in a highly selective journal like PLOS Biology.

My first concern is that the motivation of the study is overlooking decades of research that have addressed the same question as the authors. This is rather surprising given that the senior authors have themselves largely contributed to this research. Just to give a few examples, Linares et al. (2019) have presented a method to decouple sensory from decisional choice biases in perceptual decision making, Kunimoto et al. (2001) have presented behavioral results on the link between near-threshold perceptual responses and confidence judgments, and Fleming & Dolan (2012) have presented the neural bases for these behavioral results.
We appreciate the reviewers concern and it was not our intention to omit relevant studies in our article. Based on the comments of all reviewers we have now added many references to the paper that were considered relevant and important and we have discussed those studies in relation to our results. We have incorporated the suggested references by the reviewer to better motivate the study and clarify why we believe it differs substantially from previous work. We would like to note that the main goal of this study was not to reveal the neural processes underlying the computation of confidence (e.g., as in Fleming and Dolan 2012), or to dissociate perception from confidence (e.g., as in Balsdon et al., 2021, see also our response to point 3 below). In the present study confidence was not treated as a dependent measure of interest. By asking for confidence during each decision we were able to arbitrate between errors that likely reflected true perceptual illusions, when they were made with high confidence, versus errors with a less strong perceptual component, when they were made with low confidence. Confidence level was therefore merely a way to select and group specific trials for subsequent analyses. This also explains why we had initially omitted several references related specifically to confidence computations and also why we are hesitant to add new analyses related to the computation of confidence and metacognition (see point 3). We would also like to highlight that we believe neural measures are crucial in this respect to test whether the decision errors stem from sensory errors or decision errors and therefore pure behavioral approaches (like in Linares et al., 2019) may not suffice to address the questions of interest. The Linares et al. paper is indeed very relevant and we have discussed it in the introduction (e.g., page 2) as well as discussion (e.g. page 14) of the revised manuscript (reference number 9).

My second concern is that the novelty of the results is exaggerated. The authors seem surprised that early sensory processing is consistent with the physical stimulus whereas later processing is consistent with the percept. How could it be otherwise? Again, the neural basis of this hierarchical processing of ambiguous information has been established decades ago (see e.g. Logothetis & Schall, 1989).
We appreciate the reviewers comments and they have made us realize that we could have done a better job bringing across the main message, and thereby novelty, of the paper. The novelty of the paper is not so much that early sensory processing is consistent with physical stimulus properties and that later processing is consistent with the percept. We agree with the reviewer that this would not have been a sufficiently novel message for a high impact journal such as PLoS Biology. The main novelty lies in the fact that previous studies have not been able to tease apart whether relatively “late processing” observed during perceptual decision-making, and often associated with conscious perception, is merely related to the decision about the stimulus or whether it is related to perceptual, or even phenomenal properties, of the stimulus. In line with this, it remains an unresolved issue whether perceptual experience mostly depends on relatively local recurrent interactions within sensory regions or more global whole brain neural interactions (incorporating prefrontal and parietal cortex) (both “late processes”). This issue is at the heart of discussions about the neural basis of consciousness and it is highlighted in recent results as well as discussion/opinion papers in several high-impact outlets (3–7). In the present paper, we show that late processing can be subdivided in at least two subprocesses, one of which is related to the decision about a stimulus, and another that is related to perceptual (or even phenomenal) properties of a stimulus.

We believe we provide important insight in this debate, as we show that the subjectively experienced percept is represented in the same or highly similar representational format as the physical stimulus, even when the experienced percept is illusionary (that physical stimulus was not presented). We could do so, because we have designed an experiment with three crucial features: 1) two different localizer sets (sensory and decision localizers), which allows us to separate sensory from decision related multivariate EEG signals, 2) a discrimination task that yields many erroneous reports about the image that was presented (~ 30%), and 3) the inclusion of confidence reports about each decision. We show that large parts of the late EEG signal typically observed when participants report to perceive an object are in fact related to perceptual decisions, as illustrated by the comparison of Figure 3 and Figure 4 (note that the Figures numbers have changed due to new analyses and reordering of Figures in general). In these figures we show that the large square-shaped decoding profile is strongly diminished when EEG classifiers are trained on the sensory localizer and tested on the main decision task. Interestingly, doing so revealed that it’s not the typical late on-diagonal signal (reminiscent of the P3 ERP component in time-domain ERPs) that explains the difference between high and low confidence incorrect decisions, but it is the off-diagonal signal, likely reflecting longer lasting perceptual processes originating from sensory regions (8–10). This latter conclusion could only be drawn because we had classifiers that were trained on the relatively early part of stimulus processing in a task in which face/houses were task-irrelevant and unattended (between 150-200 ms). Therefore, the sensory localizer is reminiscent of a so-called no-report paradigm, but then used to train localizers instead of being used as the main task (see (6, 7, 11, 12) for examples of no-report paradigms). We have now highlighted this better in the Results and Discussion section (right from the start) of the revised manuscript where we more explicitly target the novelty of the reported findings.

*Added on page 2-3 (Introduction):*

This strategy has a similar goal as recent approaches in the field of consciousness science, in which no-report paradigms have been introduced. In studies employing such no-report paradigms, neural processing is typically compared between experimental conditions in which participants are aware of specific stimuli, but do not have a task to do and thus merely passively perceive these stimuli, versus a condition where active report is required. Contrasting these conditions, usually also including an unaware condition, is thought to separate neural signals associated with perceptual experience per se versus neural signals of post-perceptual processes (or task-relevance in the broader sense).

*Added on page 12 (Discussion):*

Another approach to isolate neural signals associated with perceptual experience from decision- and report-related processes, is the development of no-report paradigms. In such paradigms, observers may be aware or unaware of presented stimuli, but in all cases do not make perceptual judgements about them. The resulting contrasts (aware versus unaware) is then supposed to isolate perceptual experience in the absence of post-perceptual processes. Also in this line of work, it has been shown that the majority of the high-level activations in frontal and parietal cortex disappears (similar to our late square shaped decoding), and hence such activation patterns are mostly associated with post-perceptual processes and much less so with perceptual experience itself (21–26).

My third concern is that the link between confidence judgments and perceptual decisions is only superficially explored. For instance, the four-point rating scale is analyzed as just high versus low confidence, disregarding that the lowest confidence level was linked to chance perceptual performance (Figure 1B). Some analyses commonly found in the metacognition literature (for instance using meta-d'; Maniscalco & Lau, 2012) would have been welcomed. There are also interesting EEG-based analyses worth considering on this topic (e.g. Charles et al., 2013; Balsdon et al., 2021).
We again appreciate the comment and our answer here relates to point 1 above raised by the reviewer. The main goal of this study was not to reveal the neural processes underlying the computation of confidence (e.g., as in Fleming and Dolan 2012), or to dissociate perception from confidence in neural terms (e.g., as in Balsdon et al., 2021). In this study, confidence was not used as a dependent measure per se. By asking for confidence during each decision we were able to arbitrate between errors that likely reflected true perceptual illusions, when they were made with high confidence, versus errors with a less strong perceptual component, when they were made with low confidence. Confidence is therefore a way to select and group specific trials for analysis. In this study we follow a clear hypothesis driven analysis strategy, because we employ an analysis protocol that we have used previously in a study with a similar design (Weaver et al., 2019). This enabled us to restrict the number of (potential) analyses that we could perform (e.g., similar logic for the time-windows that were selected for post-hoc analyses). Because in that previous study we collapsed across the two lowest and two highest confidence levels, we have done so as well in the current study (although we show the behavioral results for all levels of confidence for completeness). There are otherwise also too few trials in each confidence condition for EEG analyses, which we knew beforehand because this was also the case in our previous study. We believe that this hypothesis driven approach is a strong feature of the article and therefore it is our preference to stick to our original plans and ideas, and therefore not split up the analyses in four confidence conditions. Further, because confidence is not a dependent measure in our design, we had not included measures that are commonly used in the metacognition literature, such as meta- d’ and do not report EEG analyses that are focused on unraveling EEG correlates of confidence, such as the ones suggested, which are very interesting and useful when serving the purpose of the study. Because we felt that adding the type of analyses that were suggested may distract the reader from the main goal and message of the paper, we have left them out.

Minor comments:
- Figures 2E, F, G, & H: would it be possible to use the same scale on the y-axis to better compare classification performance between correct and incorrect decisions?

Thanks for the suggestion. We have followed the reviewer’s suggestion and have redesigned and replotted all figures of the paper. We now plot the data between correct and incorrect decisions on the same scale and even in the same figure panels, both for the decoding line plots as well as bar plots.

- line 213: replace "Figure 3E" by "Figure 2E"
Thanks, we fixed that error.

References

Balsdon, T., Mamassian, P. & Wyart, V. (2021). Separable neural signatures of confidence during perceptual decisions. ELife, 10, e68491. <https://doi.org/10.7554/elife.68491>

Charles, L., Van Opstal, F., Marti, S., & Dehaene, S. (2013). Distinct brain mechanisms for conscious versus subliminal error detection. NeuroImage, 73, 80-94. <https://doi.org/10.1016/j.neuroimage.2013.01.054>

Fleming, S. M. & Dolan, R. J. (2012). The neural basis of metacognitive ability. Philosophical Transactions of the Royal Society of London Series B, Biological Sciences, 367(1594), 1338-1349. <https://doi.org/10.1098/rstb.2011.0417>

Kunimoto, C., Miller, J., & Pashler, H. (2001). Confidence and accuracy of near-threshold discrimination responses. Consciousness and cognition, 10(3), 294-340. <https://doi.org/10.1006/ccog.2000.0494>

Linares, D., Aguilar-Lleyda, D. & López-Moliner, J. (2019). Decoupling sensory from decisional choice biases in perceptual decision making. ELife, 8, e43994. <https://doi.org/10.7554/elife.43994>

Logothetis, N. K., & Schall, J. D. (1989). Neuronal correlates of subjective visual perception. Science, 245(4919), 761-763. <https://doi.org/10.1126/science.2772635>

Maniscalco, B. & Lau, H. C. (2012). A signal detection theoretic approach for estimating metacognitive sensitivity from confidence ratings. Consciousness and Cognition, 21(1), 422-430. <https://doi.org/10.1016/j.concog.2011.09.021>

We appreciate the suggestion of these references and we have included the majority of them in the revised manuscript.

**Rev. 3: Paul Sajda - note that this reviewer has signed his review**

In this paper, Aliliovic et al investigate how misinterpretations of sensory stimuli are reflected in EEG activity using an MVPA approach that separates sensory and decision related processes in the neural activity. They find that when subjects are confident in a decision which is an "error" (i.e. their choice does not match the stimulus category) sensory stages of stimulus processing represent the category and then later flip to a representation of the choice category. Note the reason this is important is that it is only seen for the high confidence, erroneous decisions. This work adds to the literature on how sensory evidence is transformed into a decision and how confidence may arbitrate bottom up and top down process.

I very much enjoyed the paper and I think it adds to the literature. I have some comments below that I think might help clarify the presentation a bit, particularly within the context of prior work. To be completely transparent most of the prior work I will reference below I am a co-author, and though I am asking questions related to this prior work, by no means do I require/expect the authors to reference all this work unless they believe it relevant. However there are a number of interesting findings they report that map nicely to some of my prior work that I think would be nice to reconcile/consider. I sign my name at the bottom of this review so that the authors know who I am.

1. Interaction between sensory and decision making processes explain differences between stimulus category and choice.

The interaction and "flipping" of neural representation for high confidence error trials is a very interesting finding showing the interaction between confidence, choice and sensory input. in [1] our group used a similar task (discriminating faces from houses or cars) and simultaneously recorded EEG and fMRI and showed that differences in interactions between "early" and "late" networks could explain decision bias. Note that no reports of confidence were used in our study, however bias was the measure that was representative of the mismatch between stimulus and choice category. I think that prior work and this new paper presented by the authors together show that there is clearly a bottom up and top down interaction occurring when "perceptual illusions" or "decision bias" is in play -- i.e. when the sensory category does not match the choice category and individuals are confident in their choice. It would be interesting to know the authors thoughts with respect to this prior work. Also, as an aside, did you observe any face bias for the decision errors?
We appreciate the reviewer’s positive evaluation of our results and we agree that the suggested paper (Tu et al., 2017) is highly relevant and we apologize that we omitted this study initially. We now cite the paper throughout the manuscript and specifically discuss the results on page 14 of the revised manuscript.

Regarding the question about any face bias in decision errors, we checked. In the current study we did not find an overall face bias, but more of a house bias. The proportion face vs. house responses is 42% vs 58%. In fact, in a previous study using a highly similar paradigm we found the same (Weaver et al., 2019). We have added these results to the updated manuscript on page 4.

*Added on page 14:*

Another related and interesting finding has been reported by Tu and colleagues (8). In their task, participants had to discriminate faces from houses or cars while EEG and fMRI were measured simultaneously. As the authors have observed in different task contexts (18, 84), there were both early (~200 ms after stimulus) and late (~500 ms after stimulus) EEG components that discriminated between stimulus categories. Interestingly, in behavior, participants had an overall face bias: they often misreported non-faces as faces. By combining EEG with fMRI the authors showed that individual differences in the strength of this face-bias correlated across participants with the strength of the interaction between "early" (e.g., FFA, parahipocampal place area, parietal regions) and "late" neural networks (primarily frontal regions, e.g., anterior cingulate cortex). Therefore, the strength of a face decision bias may depend on the degree of top-down predictive modulations from frontal to sensory cortices. Because no single trial confidence reports were obtained in that study, the authors could not relate their neural data and categorical responses to introspective confidence in the observers’ decisions. Our study therefore extends this previous work by showing that (at least) high confidence decision errors have a perceptual origin. In general, together these studies suggest that both perceptual biases and decision biases depend on interactions between top-down and bottom-up processes (see also (9, 12, 14, 85) for ways aiming to behaviorally disentangle sensory from decision biases).

2. Separating sensory and decision processes.

This of course is key to the work presented here. I would recommend adding a little more detail about how the MVPA was trained/optimized and also on interpretation (see below). Also some other work that might be worth considering (though once again just a suggestion) which supports these types of approaches to disassociate different processing stages in perceptual decision making.

[2] used MVPA classifiers on EEG for a face vs car task to separate sensory and evidence related signals. Together with [3] this work showed that early discriminating components are likely related to an iconic memory or "perceptual maintenance stage" (see your manuscript, line 133), and that in fact when comparing the single trial variability of these components using a DDM [4] the components clearly dissociate into a sensory evidence variable and decision/choice evidence variable. [5] uses a MVPA technique to show that multiple decision related signals are expressed across a perceptual decision and that they can be tracked via analysis of the MVPA output across time. This prior work just reinforces the approach the authors take and shows that one can use MVPA to dissociate neural correlate of perceptual and cognitive processes.

We very much appreciate that the reviewer reminded us of the suggested studies, which we regrettably omitted in the writing process. We have added references to all these studies in the revised manuscript and we have integrated this line of work at several places throughout the manuscript. We have done so while describing the rationale of the study (e.g., page 2-3), during the presentation of the results (e.g., page 4, 8 and 9) as well as in the discussion of the implications of the findings (e.g., page 11 and 12). See also the paragraph added to the discussion related to the point above.

Note that the question regarding the details of the MVPA approach are dealt with below (point 3).

3. MVPA classifier

I think it would be helpful to know a little more about the classifier. For example, is it a logistic regression classifier, LDA, etc. Was the optimization regularized? In the supplementary material it would be good to show mathematical expressions for the classifier as well as the equation for what is being optimize and if this is a convex optimization or is regularized in some way. In Fig2 and 3, panel A, plotting significant channels is interesting but it might be better to plot forward models which take into account the multivariate interactions between channels and would also be more interpretable.

We indeed employed a regularized Linear Discriminant Analysis (LDA) to perform our backward decoding. To do so, we used the standard “fitdiscr” function in Matlab where all classes have the same covariance matrix (<https://nl.mathworks.com/help/stats/fitcdiscr.html>). We have added this information to the Methods section of the paper on page 24. Because this is a relatively standard approach and it is not common to report the mathematical expression of such standard analyses we have not included these in the current version of the manuscript.

Regarding the plotting of the significant channels. Indeed, as the reviewer mentions, weights of forward models are directly interpretable, whereas the weights of backward models are not. Weights of backward models can be made interpretable however. Haufe and colleagues (13) have nicely demonstrated a transformation by which extraction filters of any linear backward model can be turned into activation patterns of a corresponding forward model, and these methods have been implemented in several MVPA toolboxes by now (including the one we used (14)). For that reason, we plotted topographic maps resulting from multiplying the data covariance matrix with the classifier weights, yielding activity patterns that are interpretable as neural sources underlying the (backward) decoding results. This approach is described on page 26 of the revised manuscript.

*On page 26:*

To inspect the pattern of neural activity that drove classification performance, we computed topographic maps for each classifier set (early, mid, late). Weights resulting from backward decoding models are not interpretable as neural sources (99). For that reason, we plotted topographic maps resulting from multiplying the data covariance matrix with the classifier weights, yielding activity patterns that are interpretable as neural sources underlying decoding results (96). These covariance/class separability maps were then normalized across electrodes for each participant (mean activity over electrodes was thus zero).

4. Minor comments

Figure 1B should be clarified. Is significance between confidence levels relative to d' =0 or is this significance relative to one another? It is clear that for confidence = 1, that this is respect to d' = 0, but not clear for the other confidence levels.

Thanks for pointing this out. The significant stars indicated the increase in perceptual sensitivity (d’) with increased level of confidence, so relative to each other (from an Anova). However, we realize that the way we plotted the stars may have been confusing, and therefore we have now omitted the stars to avoid confusion (also based on a similar comment from reviewer 1).

The sensory localizer also seems to be an oddball task (in frequent (1/5) contrast change). Perhaps mention how this will add an orienting response to the sensory localizer component. I don't think it is problematic per se, but it adds another process into what the sensory localizer represents.
Thanks for pointing this out. Indeed, participants had to indicate a contrast change of the fixation cross that happened during the interstimulus interval on 20% of the inter trial intervals. One may therefore argue this adds an orienting process to what the sensory localizer represents, although the critical stimuli (faces/houses) were task irrelevant during this task and the moment that the fixation changed in contrast did not coincide in time with the onset of the face/house stimuli. We have added this to the methods of the revised manuscript on page 22.

On page 22

Note that this change detection task adds an orienting response to the fixation cross on some trials, which was however unrelated to, and not predictive of, the occurrence of a task-irrelevant face or house stimulus.

References cited above

[1] Tu, T., Schneck, N., Muraskin, J., & Sajda, P. (2017). Network configurations in the human brain reflect choice bias during rapid face processing. Journal of Neuroscience, 37(50), 12226-12237.

[2] Philiastides, M. G., Ratcliff, R. & Sajda, P. Neural representation of task difficulty and decision making during perceptual categorization: a timing diagram. The Journal of neuroscience : the official journal of the Society for Neuroscience 26, 8965-8975 (2006).

[3] Philiastides, M. G. & Sajda, P. EEG-informed fMRI reveals spatiotemporal characteristics of perceptual decision making. The Journal of neuroscience : the official journal of the Society for Neuroscience 27, 13082-13091 (2007).

[4] Ratcliff, R., Philiastides, M. G. & Sajda, P. Quality of evidence for perceptual decision making is indexed by trial-to-trial variability of the EEG. Proceedings of the National Academy of Sciences of the United States of America 106, 6539-6544 (2009).

[5] Philiastides, M. G., Heekeren, H. R. & Sajda, P. Human Scalp Potentials Reflect a Mixture of Decision-Related Signals during Perceptual Choices. The Journal of neuroscience : the official journal of the Society for Neuroscience 34, 16877-16889 (2014).

Thanks, we have added all references to the revised manuscript.

**References used in this response letter**

1.	D. Lakens, *et al.*, Justify your alpha. *Nat. Hum. Behav.* **2**, 168–171 (2018).

2.	E. Maris, R. Oostenveld, Nonparametric statistical testing of EEG- and MEG-data. *J. Neurosci. Methods* **164**, 177–190 (2007).

3.	N. Block, What Is Wrong with the No-Report Paradigm and How to Fix It. *Trends Cogn. Sci.* **23**, 1003–1013 (2019).

4.	N. Tsuchiya, M. Wilke, S. Frässle, V. A. F. Lamme, No-Report Paradigms: Extracting the True Neural Correlates of Consciousness. *Trends Cogn. Sci.* **19**, 757–770 (2015).

5.	V. Kapoor, *et al.*, Decoding internally generated transitions of conscious contents in the prefrontal cortex without subjective reports. *Nat. Commun.* **13** (2022).

6.	S. Frassle, J. Sommer, a. Jansen, M. Naber, W. Einhauser, Binocular Rivalry: Frontal Activity Relates to Introspection and Action But Not to Perception. *J. Neurosci.* **34**, 1738–1747 (2014).

7.	E. Hatamimajoumerd, N. A. Ratan Murty, M. Pitts, M. A. Cohen, Decoding perceptual awareness across the brain with a no-report fMRI masking paradigm. *Curr. Biol.*, 1–11 (2022).

8.	S. Marti, S. Dehaene, Discrete and continuous mechanisms of temporal selection in rapid visual streams. *Nat. Commun.* **8** (2017).

9.	M. D. Weaver, J. J. Fahrenfort, A. Belopolsky, S. Van Gaal, Independent neural activity patterns for sensory-and confidence-based information maintenance during category-selective visual processing. *eNeuro* **6** (2019).

10.	R. M. Cichy, D. Pantazis, A. Oliva, Resolving human object recognition in space and time. *Nat. Neurosci.* **17**, 455–462 (2014).

11.	A. Canales-Johnson, E. Al., Directed information sharing in the brain distinguishes perception from its consequences during perceptual bistability. *Biorxiv*, 1–21 (2021).

12.	J. K. Hesse, D. Y. Tsao, A new no-report paradigm reveals that face cells encode both consciously perceived and suppressed stimuli. *Elife* **9**, 1–20 (2020).

13.	S. Haufe, *et al.*, NeuroImage On the interpretation of weight vectors of linear models in multivariate neuroimaging. *Neuroimage* **87**, 96–110 (2014).

14.	J. J. Fahrenfort, J. van Driel, S. van Gaal, C. N. L. Olivers, From ERPs to MVPA using the Amsterdam Decoding and Modeling toolbox (ADAM). *Front. Neurosci.* **12** (2018).
