# Supplementary material for: Illusory object recognition is either perceptual or cognitive in origin depending on decision confidence
Source: PLoS Biol. 2023 Mar 2;21(3):e3002009. doi: 10.1371/journal.pbio.3002009 (PMC10013920; doi:10.1371/journal.pbio.3002009)
Supplement: Attachment — Submitted filename: Rebuttal_Alilovic_2nd_revision.docx [file pbio.3002009.s008.docx]

Amsterdam, January 16th, 2023

Dear Editor, dear Kris,

We would like to express our gratitude towards the reviewers and you for the swift revision process and clear commentaries on our manuscript. We have addressed your final comments below.

We hope that we have addressed your remarks to satisfaction and hope you will now find the manuscript suitable for publication in PLOS Biology.

Yours sincerely, and on behalf of all co-authors,

Simon van Gaal

*Editorial Requests:*
Article title - Please consider a modified title that will more completely convey the interesting findings in this work. We'd suggest something like:
Illusory object recognition reflects either sensory perceptual or cognitive origins depending on decision confidence

Thanks for this title suggestion. We follow your suggestion and have changed the title to: Illusory object recognition is either perceptual or cognitive in origin depending on decision confidence (see the revised document with track changes).

Blurb – Please also provide a blurb which, if the paper is accepted, will be included in our weekly and monthly Electronic Table of Contents (eTOCs), sent out to readers of PLOS Biology. This blurb may also be used to promote your article on social media. The blurb should be about 30-40 words long and is subject to editorial changes. It should, without exaggeration, entice people to read your manuscript, should not be redundant with the title and should not contain acronyms or abbreviations. For examples, view our author guidelines: https://journals.plos.org/plosbiology/s/revising-your-manuscript#loc-blurb

We have added a Blurb, which is pasted below.

The occurrence of perceptual illusions, the misinterpretation of sensory input, is a common characteristic of several neurological and psychiatric disorders. Here we show that such illusions have both perceptual as well as cognitive neural origins in the healthy human brain.

As you address these items, please also take this last chance to review your reference list to ensure that it is complete and correct. If you have cited papers that have been retracted, please include the rationale for doing so in the manuscript text, or remove these references and replace them with relevant current references. Any changes to the reference list should be mentioned in the cover letter that accompanies your revised manuscript.

Thanks. We have checked the references and all is correct. No changes have been made.

When going over the manuscript for a final check we noticed a tiny error in the formulation of one sentence in the “participants” section on page 15 of the manuscript. We have rewritten that sentence and is thus corrected (see the revised document with track changes). Our apologies.

We note that the DOI link provided on Figshare (https://doi.org/10.21942/uva.c.6265233) is not active. This will need to be updated to allow our readership full access to the underlying summary data.

We have uploaded all data and scripts, made sure the Figshare link is active and we have ensured that readers can assess our analysis and reproduce all figures by running the code.

Note that we do not require all raw data. Rather, we ask that all individual quantitative observations that underlie the data summarized in the figures and results of your paper be made available in one of the following forms:

1) Deposition in a publicly available repository (such as Figshare or Zenodo). Please also provide the accession code or a reviewer link so that we may view your data before publication.

Done

2) Supplementary files (e.g., excel). Please ensure that all data files are uploaded as 'Supporting Information'.

In either case, please make sure the files are invariably referred to (in the manuscript, figure legends, and the Description field when uploading your files) using the following format verbatim: S1 Data, S2 Data, etc. Multiple panels of a single or even several figures can be included as multiple sheets in one excel file that is saved using exactly the following convention: S1_Data.xlsx (using an underscore).

***Regardless of the method selected, please ensure that you provide the individual numerical values that underlie the summary data displayed in the following figure panels as they are essential for readers to assess your analysis and to reproduce it:
Fig 1B; Fig 2A,B; Fig 3A-E; Fig 4A-E; Fig 5A-C; SuppFig 2A-F; SuppFig 3; SuppFig 4A-C; SuppFig 5A-C

Done

***Please also ensure that figure legends in your manuscript and in the supplemental file are updated to include information on WHERE THE UNDERLYING DATA CAN BE FOUND (e.g. “The underlying data supporting Fig X, panel Y can be found in file Z.”). Please also ensure that your supplemental data file/s has a legend.

We have added statements in the figure legends about where the data and scripts can be found for each figure.


Please ensure that your Data Statement in the submission system accurately describes where your data can be found, with the DOI link being updated to an active file link.

Done


------------------------------------------------------------------------

Reviewer remarks:

Reviewer's Responses to Questions

Do you want your identity to be public for this peer review?

Reviewer #1: Yes: Guillaume Rousselet

Reviewer #2: No

Reviewer #3: Yes: Paul Sajda


Reviewer #1: The authors have tackled all my comments. I've also read the reply to the other reviewers and I think the new version addresses all their comments as well. Emphasis on novelty is not something I consider important, but the authors have now clearly articulated how their work builds upon previous studies on a similar topic. The revision made an already strong article even stronger: the writing is sharper, the reporting and figures more detailed and informative, and the interpretations more nuanced. I would change the wording of a few things, especially in the results, but that's down to personal preferences, so no need to make any more changes at this stage. The authors should be really proud of what they have achieved here.


Reviewer #2: I would like the authors for taking all the reviewers' comments seriously, including mine, and for writing a detailed response letter. The changes made to the manuscript in the text and the figures are very much welcomed. The analogy to the no-report paradigm definitely helped me clarify a few points.

On the use of confidence reports, the authors are stating that "confidence was not used as a dependent measure per se" (response letter), but in the abstract, they admit "This work demonstrates that decision confidence arbitrates between perceptual decision errors, which reflect true illusions of perception, and cognitive decision errors, which do not". So it seems that confidence was after all an important dependent variable in their design. However, I agree with the authors that an in-depth analysis of this variable would have made this manuscript much longer and more cumbersome.

In summary, I think the results are clean, the analyses are trust-worthy, and thus this manuscript deserves to be nicely published.

Reviewer #3: The reviewers have addressed all my comments and concerns and I recommend the manuscript be accepted.

Signed: Paul Sajda
